# Comparison of the Sixth and the Seventh Editions of the UICC Classification for Perihilar Cholangiocarcinoma

**DOI:** 10.1245/s10434-012-2486-0

**Published:** 2012-07-18

**Authors:** Benjamin Juntermanns, Georgios Charalambos Sotiropoulos, Sonia Radunz, Henning Reis, Matthias Heuer, Hideo Andreas Baba, Ali Canbay, Martin Schuler, Guido Gerken, Andreas Paul, Gernot Maximilian Kaiser

**Affiliations:** 1 Department of General, Visceral and Transplantation Surgery, West German Cancer Center, University Hospital Essen, University Duisburg-Essen, Essen, Germany; 2 Department of Pathology and Neuropathology, West German Cancer Center University Hospital Essen, University Duisburg-Essen, Essen, Germany; 3 Department of Gastroenterology and Hepatology, West German Cancer Center, University Hospital Essen, University Duisburg-Essen, Essen, Germany; 4 Department of Medical Oncology, West German Cancer Center, University Hospital Essen, University Duisburg-Essen, Essen, Germany

## Abstract

**Background:**

The seventh edition of the TNM classification separates extrahepatic bile duct tumors into perihilar and distal tumors and further changes the definition of the TNM classification. The impact of the seventh edition on stage-based prognostic prediction for patients with perihilar cholangiocarcinoma was evaluated.

**Methods:**

Between January 1998 and March 2010, 223 consecutive patients with perihilar cholangiocarcinoma underwent surgery at the West German Cancer Center. Median survival times were calculated for the 195 evaluable patients (excluding those with in-hospital mortality) after separate classification by both sixth and seventh editions.

**Results:**

Median overall survival was increased in patients classified using the seventh compared with the sixth edition (UICC I: 56.5 vs 23.75 months; II: 45.9 vs 31.6 months; III: 21.3 vs. 8.76 months; IV: 7.03 vs 5.93 months). The T category of the seventh edition did not alter median survival times of T1 (54.07 months) and T4 (7.83 months) cases, but median survival was prolonged for T2 patients (29.4 vs 31.6 months), and shortened for T3 patients (19.43 vs 11.8 months) staged using the seventh edition. According to Cox proportional hazards regression analysis, patient survival was better predicted by the seventh edition UICC stage and pT categories (*p* = 0.0014 and *p* = 0.0396, respectively), than the corresponding sixth edition categories (*p* = 0.4376 and *p* = 0.0926, respectively).

**Conclusions:**

The UICC seventh edition TNM classification for perihilar cholangiocarcinoma improves separation of patients with intermediate stage tumors compared with the sixth edition. The prognostic value of the UICC staging system has been strengthened by the introduction of the seventh edition.

With only 2–4 new cases per 100,000 people per year, the hilar cholangiocarcinoma is an uncommon malignant tumor, but is the fourth most common gastrointestinal malignancy.^1^^,^ 2 Surgical treatment of hilar cholangiocarcinoma comprises extrahepatic bile duct resection, hepatic resection, vascular resection, and lymph node dissection. This strategy is associated with up to 19 % patient mortality and perioperative morbidity from 14 to 76 %.^3^ These higher morbidity and mortality rates are observed as a result of the necessity of more extensive hepatic resection combined with resection of the extrahepatic bile duct.^4^^,^ 5 Recent studies report 5-year survival following complete surgical resection of the perihilar cholangiocarcinoma combined with major hepatic resections in the range of 25–40 %.^3^^,^ 6 In addition to resection, liver transplantation may also offer a curative treatment option for selected patients suffering from hilar cholangiocarcinoma.^7^^–^ 9 The UICC TNM classification aims to reflect the outcome of patients with perihilar cholangiocarcinoma.^3^^,^ 10 ^–^ 12 The sixth edition was published in 2002 and primarily relied on the presence of lymph node metastasis and the extent of vascular invasion, the latter requiring vascular resection and reconstruction in this tumor entity.^13^ The seventh edition, published in 2009, further separates extrahepatic cholangiocarcinoma into two groups by either perihilar (proximal) or distal localization of the tumor.^14^ Interestingly, T3 stage of the sixth edition included tumors infiltrating neighboring organs, such as the gall bladder, pancreas, or the liver parenchyma. A tumor infiltrating the duodenum was classified as T4. In contrast, perihilar cholangiocarcinoma infiltrating neighboring organs such as the duodenum, but not the hepatic parenchyma, is not clearly defined by the seventh edition. Cases with regional lymph node metastases have also been reclassified in the seventh edition. In particular, tumors spreading into celiac and superior mesenteric lymph nodes, which were staged as N1 by the sixth edition, are classified as M1 by the seventh UICC edition. These changes result in the reclassification of former UICC Stage IIB tumors (sixth edition) as UICC stage IVB tumors (seventh edition) if lymph node metastases were not regional.

A staging system that more exactly separates patients suffering from hilar cholangiocarcinoma into prognostic groups is desirable to support patient stratification for treatment in light of future multimodal perioperative therapeutic strategies. Clinical staging of perihilar cholangiocarcinoma prior to surgery is challenging since computed tomography (CT) or magnetic resonance imaging frequently fails to define the full extent of the tumor. Dual-modality PET/CT imaging has been shown to detect metastases of hilar cholangiocarcinoma in lymph nodes and other distant locations with high specificity.^15^ In addition, expression of biomarkers such as vascular endothelial growth factor A and metallothionein has been shown to correlate with survival of patients suffering from extrahepatic cholangiocarcinoma.^16^^,^ 17 In a recent report, patients homozygous for the C allele of the GNB3 825C>T single nucleotide polymorphism exhibited a significantly prolonged survival compared with patients heterozygous for this polymorphism or lacking the C allele.^18^ However, the prognostic value of these markers will have to be prospectively confirmed before they can be applied to patient selection for adjuvant therapy regimens.

Against this background, we compared the sixth and seventh editions of the UICC TNM classification for perihilar cholangiocarcinoma in 223 patients consecutively treated at our center over a 12-year period. The aim of this study was to investigate whether classification of perihilar cholangiocarcinoma according to the seventh TNM edition provides better differentiation between tumor stages and more accurately predicts patient survival.

## Patients and methods

### Patients

Between January 1998 and March 2010, 247 patients with the suspected diagnosis of hilar cholangiocarcinoma were surgically treated at our center. Routine histopathological workup was conducted for all resected tumors by the Department of Pathology and Neuropathology of the University Hospital Essen. Benign conditions in accordance with a Klatskin-mimicking lesion were diagnosed in 24 patients.^19^ Cholangiocellular adenocarcinoma was diagnosed in 223 patients, including 128 male (57 %) and 95 female (43 %) patients, with a mean age of 61 (*±*11) years. All types of resection margins (R0, R1, and R2) and all cases of irresectable disease were included in the study cohort. Patients with postoperative in-hospital mortality (28 patients) were excluded from further evaluation to focus on malignancy-related outcome. Thus, a total of 195 patients were available for evaluation.

### Histopathological Processing

Surgical specimens were stored in 4 % neutral-buffered formalin (12–24 h) prior to histopathological processing, then dehydrated and cleared using an automated standard procedure (Shandon Pathcentre, Thermo Fisher Scientific Inc., USA) before paraffin embedding in Paraplast (McCormick Scientific, USA). From each paraffin block, 3–5 μm sections were prepared (Leica SM2000R, Leica Microsystems, Germany) and mounted on glass slides. Hematoxylin and eosin (H&E; Merck, Germany; Chroma/Waldeck, Germany) staining was performed following standard diagnostic procedures (Shandon Varistain Gemini, Thermo Fisher Scientific Inc., USA). Histopathology reports were available for every case and included macroscopic and microscopic tumor evaluations, a continuous text summary, and the TNM classification. Data including operative reports and surgical pathology reports of all patients were entered prospectively into a computer database. Cases were stratified according to the UICC staging system and TNM classifications based on the “Extrahepatic Bile Duct” chapter in the sixth edition and the new “perihilar cholangiocarcinoma” chapter in the seventh edition.^13^^,^ 14

### Data Analysis

Changes in the distribution of TNM classifications and UICC stages were compared between the sixth and seventh editions, and median survival and survival ranges were calculated independently for each classification. Additionally, 1-, 3-, and 5-year survival rates were calculated. Survival was calculated using the Kaplan–Meier method and the log-rank test. Cox proportional hazards regression analysis was performed to evaluate the prognostic value of the TNM categories and UICC stages derived from the sixth and seventh editions. Differences of *p* < 0.05 were considered to be statistically significant. Statistical analyses were performed using JMP statistical software, version 8.0.2 (SAS, Cary, NC).

## Results

A total of 195 patients suffering from perihilar cholangiocarcinoma were surgically treated at our center between January 1998 and March 2010. In this retrospective study, we compared the impact of applying either the sixth or seventh editions of UICC tumor staging to stratify median patient survival or predict prognosis in this patient cohort. Cases of postoperative in-hospital mortality were excluded from the analysis. A summary of differences between the sixth and seventh editions of UICC staging of extrahepatic bile duct tumors and the respective TNM categories is presented in Tables 1 and 2.^13^^,^ 14 We compared the influence of tumor staging using either the sixth or seventh UICC editions on the 1-, 3-, and 5-year survival of patients in this cohort (Table 3). The median overall survival for patients staged according to the sixth or seventh editions and broken down by tumor stage were: stage I (23.75 or 56.5 months), stage II (31.6 or 45.9 months), stage III (8.76 or 21.3 months), and stage IV (5.93 or 7.03 months), respectively. Staging according to the seventh edition resulted in an increased median overall survival for patients suffering from perihilar cholangiocarcinoma throughout all tumor stages, and a change in staging occurred in 92 patients.

**Table 1:** UICC stages according to the sixth and seventh editions of the TNM classification

UICC staging system							
Sixth edition				Seventh edition			
Stage	TNM	N	M	Stage	TNM	N	M
0	Tis	N0	M0	0	Tis	N0	M0
Ia	T1	N0	M0	I	T1	N0	M0
Ib	T2	N0	N0	–	–	–	–
IIa	T3	N0	M0	II	T2a,b	N0	M0
IIb	T1–3	N1	M0	–	–	–	–
III	T4	AnyN	M0	IIIa	T3	N0	M0
–	–	–	–	IIIb	T1–3	N1	M0
IV	T1–4	AnyN	M1	IVa	T4	AnyN	M0
–	–	–	–	IVb	T1–4	AnyN	M1

**Table 2:** TNM categories according to the sixth and seventh editions of the TNM classification

Sixth edition		Seventh edition	
T category			
Tx	Primary tumor cannot be assessed	Tx	Primary tumor cannot be assessed
T0	No evidence of primary tumor	T0	No evidence of primary tumor
Tis	Carcinoma in situ	Tis	Carcinoma in situ
T1	Tumor confined to the bile duct	T1	Tumor confined to the bile duct, with extension up to the muscle layer or fibrous tissue
T2	Tumor invades beyond the wall of the bile duct	T2a	Tumor invades beyond the wall of the bile duct to surrounding adipose tissue
–	–	T2b	Tumor invades adjacent hepatic parenchyma
T3	Tumor invades the liver, gall bladder, pancreas, and or unilateral tributaries of the portal vein (right or left) or hepatic artery (right or left)	T3	Tumor invades unilateral branches of the portal vein or hepatic artery
T4	Tumor invades any of the following: main portal vein or its tributaries bilaterally, common hepatic artery, or other adjacent structures, e.g., colon, stomach, duodenum, abdominal wall	T4	Tumor invades the main portal vein or its branches bilaterally; or the common hepatic artery; or the second-order biliary radicals bilaterally; or unilateral second-order biliary radicals with contralateral portal vein or hepatic artery involvement
N category			
Nx	Regional lymph nodes cannot be assessed	Nx	Regional lymph nodes cannot be assessed
N0	No regional lymph node metastasis	N0	No regional lymph node metastasis
N1	Regional lymph node metastasis are the cystic duct, pericholedochal, hilar, peripancreatic (head only), periduodenal, periportal, celiac, and superior mesenteric nodes	N1	Regional lymph node metastasis including nodes along the cystic duct, common bile duct, common hepatic artery, and portal vein
M category			
Mx	Distant metastasis cannot be assessed	Mx	Distant metastasis cannot be assessed
M0	No distant metastasis	M0	No distant metastasis
M1	Distant metastasis	M1	Distant metastasis

**Table 3:** Median survival by UICC stage (*n* = 195) using the sixth and seventh editions of the TNM classification

UICC	*N* (%)	Median survival in months (range)	Log-rank test (*p* value)	Cox regression analysis (*p* value)	1-Year survival (%)	3-Year survival (%)	5-Year survival (%)
Sixth edition			<0.0001	0.0926			
I	26 (13.3)	23.75 (86.73–0.5)			92	61	61
II	88 (45.1)	31.6 (138.97–1.8)			72	47	31
III	22 (11.3)	8.76 (35.47–1.57)			41	0	0
IV	59 (30.3)	5.93 (60.6–0.23)			24	4	4
Seventh edition			<0.0001	0.0396			
I	6 (3.1)	56.5 (80.07–22.3)			100	80	80
II	52 (26.7)	45.9 (138.97–0.5)			79	61	44
III	51 (26.1)	21.3 (103.93–1.7)			66	36	24
IV	86 (44.1)	7.03 (60.6–0.23)			32	4	2

Survival was calculated using the Kaplan–Meier method and compared using the log-rank test. Cox proportional hazards regression analysis was performed to evaluate the prognostic value of the UICC stage according to the sixth and seventh editions

**Fig. 1:**
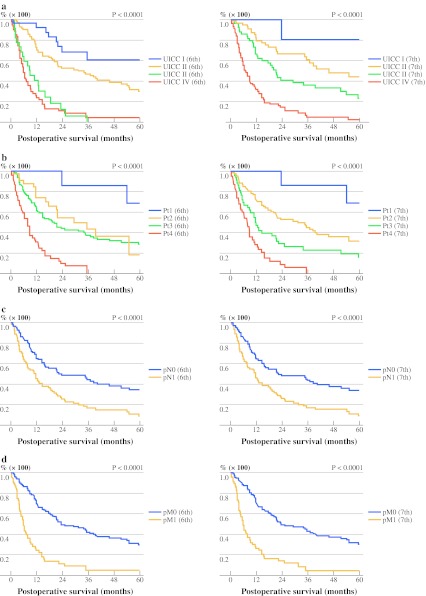
Comparison of survival prediction for perihilar cholangiocarcinoma (*n* = 195) after surgery using the sixth (*left*) and seventh (*right*) editions of the UICC tumor classification. Kaplan–Meier analysis was based on tumor stage (**a**), T category (**b**), N category (**c**), and M category (**d**). Significant differences (*p* values) in survival were assessed using the log-rank test

We also compared the ability of the sixth and seventh editions of the UICC classification to accurately predict patient prognosis based on tumor stage. Kaplan–Meier survival analysis for patients with different tumor stages revealed that the seventh edition more accurately stratifies this patient cohort according to stage (Fig. 1). This is particularly evident for patients with stage II and III tumors. The prognostic value of the seventh edition tumor staging was confirmed by Cox proportional hazards regression analysis, which was significant for the seventh edition (*p* = 0.0396), but did not reach the significance cutoff for the sixth edition (*p* = 0.0926, Table 3). Analysis of our patient cohort shows that the seventh edition improves accuracy of tumor stage classification for prognostic prediction.

The extent of tumor infiltration as reflected by the T category has been defined more specifically by the seventh edition. We compared median survival of patients based on either the sixth or seventh edition T category classification (Table 4). The seventh edition definition of the T category had no impact on median survival of patients with tumors classified as T1 (54.07 months) or T4 (7.8 months). However, the seventh edition definition of the T category resulted in slightly increased median survival in the T2 category (29.4 vs 31.6 months), but in reduced median survival in patients allocated to the T3 category (19.4 vs 11.8 months). The prognostic value of the seventh edition T category staging was confirmed by Cox proportional hazards regression analysis (*p* = 0.0014, Table 4). This analysis also showed that the sixth edition T category staging did not significantly influence prognosis (*p* = 0.4376). The more exact definition of tumor infiltration by the seventh edition T category clearly improved patient stratification particularly for intermediate tumor stages.

**Table 4:** Median survival by TNM categories (*n* = 195) using the sixth and seventh editions of the TNM classification

UICC	*N* (%)	Median survival in months (range)	Log-rank test (*p* value)	Cox regression analysis (*p* value)	1-Year survival (%)	3-Year survival (%)	5-Year survival (%)
Sixth edition			<0.0001	0.4376			
T1	8 (4.1 %)	54.07 (80.07–22.3)			100	86	69
T2	32 (16.4 %)	29.4 (86.73–0.5)			74	43	38
T3	97(49.7 %)	19.43 (138.97–0.9)			62	38	29
T4	58(29.7 %)	7.83 (35.47–0.23)			31	0	0
Seventh edition			<0.0001	0.0014			
T1	8 (4.1 %)	54.07 (80.07–22.3)			100	86	69
T2	93 (47.7 %)	31.6 (138.97–0.5)			72	45	32
T3	42 (21.5 %)	11.8 (90.33–1.8)			47	23	17
T4	52 (26.7 %)	7.83 (35.47–0.23)			28	0	0
Sixth edition			<0.0001	0.2252			
N0	108 (55.4 %)	22.43 (0.5–138.97)			65	44	34
N1	87 (44.6 %)	11.06 (0.23–103.97)			45	17	9
Seventh edition			<0.0001	0.4940			
N0	109 (55.9 %)	22.43 (0.5–138.97)			64	44	34
N1	86 (44.1 %)	11.56 (0.23–103.97)			46	17	9
Sixth edition			<0.0001	0.3432			
M0	136 (69.7 %)	23.6 (0.5–138.97)			68	42	29
M1	59 (30.3 %)	5.93 (0.23–60.6)			24	4	4
Seventh edition			<0.0001	0.2224			
M0	132 (67.7 %)	23.6 (0.5–138.97)			72	42	31
M1	63 (32.3 %)	5.93 (0.23–60.6)			26	4	4

Survival was calculated using the Kaplan–Meier method and compared using the log-rank test. Cox proportional hazards regression analysis was performed to evaluate the prognostic value of the TNM categories according to the sixth and seventh editions

Lymph nodes positive for cancer cells can either be defined as regional spreading of the tumor or as metastases. The definition of regional lymph nodes has been restricted within the N and M categories of the seventh edition of the UICC classification. The overall impact of a positive lymph node on the extent of the disease is also reflected by the seventh edition, such that involvement of any lymph node results in tumor stage III. Applying the seventh edition resulted in reclassification of four patients as M1 based on positive lymph node histopathology (Table 5). Median survival for patients classified for the N category using the sixth and seventh edition was also compared (Table 4). Median survival of patients classified N0 (22.4 months) and N1 (11.067 vs 11.567 months) largely remained unchanged. Using the sixth or seventh edition descriptions of the M category also did not affect median survival of patients either staged M0 or M1 (23.6 and 5.93 months, respectively). As expected by the minimal differences in median overall survival, Cox proportional hazards regression analysis showed no differences in the prognostic value for N and M categories between the sixth and seventh edition (Table 4).

**Table 5:** Patients with lymph node metastasis upstaged from N1 in sixth edition of the UICC TNM classification to M1 in the seventh edition of classification of regional lymph node metastasis

Patients with positive lymph nodes	Location of lymph node infiltration	Sixth TNM edition	Seventh TNM edition
Male, 54 years	Parapancreatic paracaval	N1	M1
Female, 55 years	Hepaticoduodenal	N1	M1
Male, 46 years	Celiac artery	N1	M1
Female, 54 years	Celiac artery	N1	M1

The group of extrahepatic bile duct tumors as described in the sixth edition of the UICC classification has been separated into “perihilar” and “distal” bile duct tumors by the seventh edition.^13^^,^ 14 In our cohort, 21 of 195 patients were inflicted with perihilar cholangiocarcinoma that directly infiltrated adjacent organs. Of these patients, 12 had tumor infiltration into the gallbladder, pancreas, or duodenum with additional vascular infiltration and were thus classified as either T3 or T4 by the sixth edition. The tumors from nine patients showed no vascular infiltration, but infiltrated either into the gall bladder or pancreas and were classified as T3 or the duodenum and were classified as T4 using the sixth edition.^13^ The seventh edition of the TNM classification makes no provisions for classifying tumors infiltrating adjacent organs without vascular infiltration, which accounted for 4.6 % of cases in our patient cohort. The section editor for the upper gastrointestinal tract at the UICC advised us to apply the seventh edition TNM classification schema for distal extrahepatic bile duct tumors for this group, which classifies all of these patients as T3 and T4 (adjacent organs), respectively.^14^ Withdrawal of these patients from our analysis did not considerably alter median survival (data not shown). Nevertheless, the presence of a small fraction of patients with tumors infiltrating adjacent organs but not the vasculature warrants the inclusion of this group in future editions of the TNM classification for proximal extrahepatic bile duct tumors.

## Discussion

In this retrospective, single-institution study, we show that the seventh edition of the UICC TNM classification for perihilar cholangiocarcinoma more accurately represents the severity of the disease. Particularly, the more detailed guidelines for defining the tumor extension, the higher impact attributed to lymph nodes infiltrated by tumor cells, and the separation of perihilar from distal bile duct tumors have improved the separation of clinical course and prognostic prediction based on tumor stage alone. The seventh edition constricts the definition of regional lymph node involvement in the N and M classifications and puts more weight on the presence of any lymph node infiltrated with cancer cells in tumor staging. This resulted in the reclassification of cases with lymph node metastases as UICC stage III by the seventh edition (formerly stage II in the sixth edition). In addition, stage IV has been subdivided into local invasion (IVA) and distant disease (IVB). Applying the seventh edition of the UICC staging system, we observed considerable stage migration within our patient cohort. For example, because of the exclusion of tumors infiltrating beyond the ductal wall (T2), only six patients of the 26 patients who were classified as stage I by the sixth edition were classified as such according to the seventh UICC edition. The 88 cases classified as stage II tumors according to the sixth edition was reduced to 52 cases using the seventh edition, since nodal positive (N1) tumors were excluded from stage II. In consequence, the number of cases classified as stage III according to the seventh edition increased to 51 patients (compared with 22 using the sixth edition), even though patients with T4 tumors were removed from this group by the seventh edition. Patients with T4 tumors are included in stage IV by the seventh edition, thus increasing the number of stage IV patients in our cohort from 59 (sixth edition) to 83. The four patients categorized as stage III according to the sixth edition of the TNM classification due to celiac or mesenteric lymph node infiltration (N1) were upstaged to metastatic (M1, stage IV) disease according to the seventh edition (Table 5). This stage migration based on the seventh edition of the TNM classification resulted in a better separation of the clinical course, as reflected by the higher median survival of stage I patients (56.5 vs 23.75 months) and the lower median survival of stage II and III patients (31.6 vs 45.9 and 8.7 vs 21.3 months, respectively) in our cohort.

The seventh edition of the UICC TNM classification for extrahepatic bile duct tumors, for the first time separates this group into “perihilar” and “distal” bile duct tumors. Tumors confined to the bile duct remain classified as T1 in the seventh edition, but margins for tumors infiltrating adjacent tissues have been more specifically defined. T2 now includes a new subcategory for tumors invading the adjacent hepatic parenchyma (T2b). T3 tumors have unilateral vascular invasion, and T4 is defined on the basis of bilateral biliary and/or vascular invasion, as it has been by the sixth edition. In our cohort, survival of patients categorized as T1 or T4 according to the seventh edition was unchanged from that using the sixth edition. Patient survival in our cohort was slightly increased for T2 (29.4 vs 31.6 months) cases and decreased for T3 (19.4 vs 11.8 months) cases using the seventh edition. T3 previously included tumors with continuous infiltration into neighboring organs or tissues, such as the gallbladder, pancreas, or liver parenchyma. The seventh edition classifies a tumor with continuous infiltration of the adjacent liver parenchyma as T2b. However, perihilar cholangiocarcinoma infiltrating neighboring organs can no longer be classified using the seventh TNM classification. Our cohort included 21 such patients, with perihilar cholangiocarcinoma microscopically infiltrating adjacent organs as a continuation of the primary tumor. Communicating our observation with the UICC we were advised to apply the seventh edition parameters for staging of distal extrahepatic cholangiocarcinoma to these cases.^14^ In doing so, adjacent invasion of the liver parenchyma was classified as T2b and unilateral vascular involvement as T3. Infiltration of the liver parenchyma alone, without accompanying vascular infiltration, has been associated with a better prognosis than when vascular invasion was additionally present.^20^ Therefore, the “downgraded” classification of these tumors as T2b by the seventh edition appears justified. Because of their significant representation in our large patient cohort, we propose to include an adjacent tumor infiltration classification also for perihilar as well as distal cholangiocarcinoma in future amendments to the TNM classification.

In conclusion, based on this retrospective study of 195 evaluable patients treated for perihilar cholangiocarcinoma at a single institution, the categorization of tumor stages by the seventh edition of the TNM classification improves on that by the sixth edition. The seventh edition subdivides malignant extrahepatic bile duct tumors in perihilar and distal groups and attributes tumor infiltration of lymph nodes more impact on the extent and, thus, severity of the disease. The seventh edition better separates intermediate stage tumors as reflected by the median patient survival for this cohort and confers a higher prognostic value to the tumor stage. This should facilitate stratification of patients diagnosed with perihilar cholangiocarcinoma into different risk groups that might benefit from multimodal perioperative treatment strategies. Our analyses show that particularly the staging and T categories of the seventh edition result in better prediction of patient survival than the corresponding categories from the sixth edition. Based on the considerable number of patients in our cohort whose disease was differently staged by the sixth or the seventh editions of TNM classification, previous studies using the sixth edition for staging should reconsider the changes of the seventh edition prior to comparison with new data emerging from treatment of patients with perihilar cholangiocarcinoma.
